# The effect of microvascular decompression of the CN IX-X root entry/exit zone and the ventrolateral medulla in neurogenic hypertension involving the vertebral/basilar artery

**DOI:** 10.3389/fneur.2024.1376019

**Published:** 2024-06-18

**Authors:** Defeng Zeng, Shiyao Wang, Xingrong Wei, Shuguang Zhang, Hao Zhou, Xueqian Hu, Xin Fu, Yang Li, Zhenqing Wei

**Affiliations:** ^1^Department of Neurosurgery, The First Affiliated Hospital of Dalian Medical University, Dalian, China; ^2^Department of Graduate School, Dalian Medical University, Dalian, China; ^3^Department of Neurology, The First Affiliated Hospital of Dalian Medical University, Dalian, China

**Keywords:** neurogenic hypertension, microvascular decompression, rostral ventrolateral medulla, root entry zone, vertebral/basilar artery

## Abstract

**Introduction:**

Neurogenic hypertension (HTN) is a type of HTN characterized by increased activity of the sympathetic nervous system. Vascular compression is one of the pathogenic mechanisms of neurogenic HTN. Despite Jannetta's solid anatomical and physiological arguments in favor of neurogenic HTN in the 1970's, the treatment for essential HTN by microvascular decompression (MVD) still lacks established selection criteria. Therefore, the subjects selected for our center were limited to patients with primary trigeminal neuralgia (TN) and primary hemifacial spasm (HFS) of the vertebral/basilar artery (VA/BA) responsible vessel type coexisting with neurogenic HTN who underwent MVD of the brainstem to further explore possible indications for MVD in the treatment of neurogenic HTN.

**Methods:**

A retrospective analysis of 63 patients who were diagnosed with neurogenic HTN had symptoms of HFS and TN cranial nerve disease. Patients were treated at our neurosurgery department from January 2018 to January 2023. A preoperative magnetic resonance examination of the patients revealed the presence of abnormally located vascular compression in the rostral ventrolateral medulla (RVLM) and the root entry zone (REZ) of the IX and X cranial nerves (CN IX- X).

**Results:**

There was no significant difference between the two groups in terms of gender, age, course of HFS, course of TN, course of HTN, degree of HTN, or preoperative blood pressure. Based on the postoperative blood pressure levels, nine out of 63 patients were cured (14.28%), eight cases (12.70%) showed a marked effect, 16 cases (25.40%) were effective, and 30 cases were invalid (47.62%). The overall efficacy was 52.38%. However, 39 cases of combined cranial nerve disease were on the left side of the efficacy rate (66.67%) and 24 cases of combined cranial nerve disease were on the right side of the efficacy rate (29.16%).

**Discussion:**

Over the last few decades, many scholars have made pioneering progress in the clinical retrospective study of MVD for neurogenic hypertension, and our study confirms the efficacy of MVD in treating vertebral/basilar artery-type neurogenic hypertension by relieving the vascular pressure of RVLM. In the future, with the development and deepening of pathological mechanisms and clinical observational studies, MVD may become an important treatment for neurogenic hypertension by strictly grasping the surgical indications.

**Conclusion:**

MVD is an effective treatment for neurogenic HTN. Indications may include the following: left-sided TN or HFS combined with neurogenic HTN; VA/BA compression in the left RVLM and REZ areas on MRI; and blood pressure in these patients cannot be effectively controlled by drugs.

## 1 Introduction

Hypertension (HTN) is a common cardiovascular disease and an important risk factor for chronic cardiovascular diseases such as stroke, myocardial infarction, and heart failure. Prevention and treatment of HTN can lead to a reduction in morbidity and mortality from cardiovascular diseases. According to the WHO data (1), ~1.28 billion adults worldwide aged 30–79 years suffer from HTN, most of whom (approximately two-thirds) live in low- and middle-income countries. From 1990 to 2019, the number of hypertensive patients aged 30–79 years doubled (2). It has been suggested that the activation of the sympathetic system maybe closely related to HTN (3). Neurogenic HTN is a specific type of HTN, which accounts for ~10–15% of all cases of primary HTN. In 1979, Jannetta et al. first proposed that the etiology of neurogenic HTN could be due to the presence of abnormal vascular compression in the rostral ventrolateral medulla (RVLM) area and the cephalic root entry zone (REZ) of the medulla oblongata, which leads to elevated blood pressure (4). The pathogenesis of neurogenic HTN is mainly related to the overactivity of the sympathetic nervous system (5, 6). Several studies have shown that neurogenic HTN usually refers to HTN caused by vascular compression of the RVLM and the REZ of the IX and X cranial nerves (7–12). Most hypertensive patients can achieve ideal blood pressure control with systematic and regular medication. However, some patients still have difficulty controlling their blood pressure effectively after the combined use of antihypertensive drugs. Therefore, surgical treatment of neurogenic hypertension (HTN) has opened up a whole new avenue for HTN treatment (4, 13, 14). In recent years, microvascular decompression (MVD) has been gradually introduced as a surgical treatment for neurogenic HTN. MVD relieves cranial nerve disease by isolating the causative responsible vessel from the primary cranial nerve disease and relieving the compression of blood vessels and nerves. Geiger et al. (15) performed MVD in eight patients with neurogenic HTN and found that 3 months after the procedure, seven patients had a significant decrease in blood pressure values compared to the preoperative values. Therefore, MVD for neurogenic HTN may be a recognized treatment option. However, previous investigators exploring the efficacy of MVD in the treatment of neurogenic HTN did not specifically categorize the vessels compressed in the RVLM region, and patients with basilar artery-responsible vessels required the use of “bridging” multipoint spacer pads during the procedure. That is, a cotton spacer pad is routinely placed between the RVLM area and the VA/BA. Consequently, we performed MVD on 63 patients with neurogenic HTN, cranial nerve disease, and VA/BA liability vessels from January 2018 to January 2023. While resolving the cranial nerve disorders, blood pressure levels were significantly reduced in some patients. This study provides a retrospective analysis by other scholars on the efficacy of MVD in the treatment of neurogenic HTN.

## 2 Information and methods

### 2.1 Clinical information

**Figure 1:**
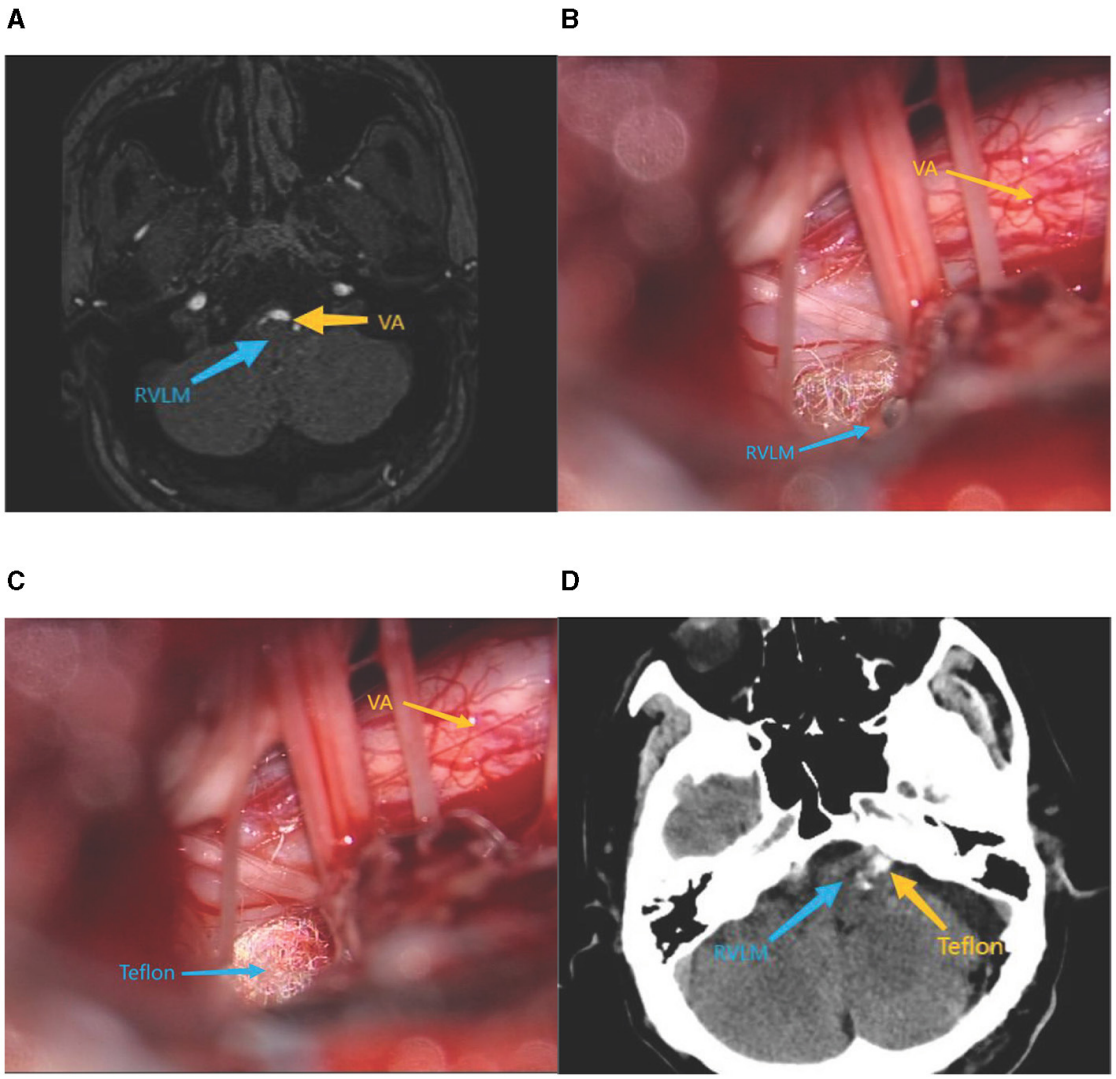
**(A)** Preoperative cranial MRI. **(B)** Intraoperative exposure of RVLM. **(C)** Intraoperative placement of Teflon pads. **(D)** Postoperative cranial ct.

A total of 63 patients with primary VA/BA type TN, HFS, and a concomitant diagnosis of neurogenic HTN were admitted to the Department of Neurosurgery of the First Affiliated Hospital of Dalian Medical University from January 2018 to January 2023. The study included 31 male patients, 32 female patients, 35 patients with TN, and 28 patients with HFS. The combined cranial nerve disease affected 39 cases on the left side and 24 cases on the right side. The age of the patients ranged from 39 to 84 years (62.02 ± 10.41). Endocrine HTN, such as primary aldosteronism and cortisolism, was excluded in all patients, as were renal HTN, renal vascular disease, chronic obstructive sleep apnea hypoventilation syndrome, and other causes of common secondary HTN. All patients had a history of HTN between 1 and 30 years with unsatisfactory blood pressure control after a long-term combination of ≥3 of 5 antihypertensive drugs (calcium antagonists, diuretics, angiotensin-converting enzyme inhibitors, angiotensin receptor blockers, and beta receptor antagonists). The observation is based on the diagnostic criteria for HTN in the Chinese HTN Prevention and Control Report 2023 (the blood pressure of patients was measured at ~6:00 a.m. and 6:00 p.m., and the mean value was taken at the time of no seizure of cranial neurological disease). According to the criteria for diagnosis of HTN in China, the definition of HTN is defined as a baseline systolic pressure >140 mmHg and a baseline diastolic pressure >90 mmHg. All patients were admitted to the hospital after posterior cranial fossa magnetic resonance thin-scanning and three-dimensional time-of-flight magnetic resonance angiography (3D-TOF-MRA). The results demonstrate the presence of abnormally located vascular compression in the RVLM and the REZ of the IX and X cranial nerves (as evidenced by the presence of vascular indentation in the RVLM and the presence or change in the location of vascular compression in the REZ of the IX and X cranial nerves), as shown in Figure 1A.

### 2.2 Surgical method

All patients who underwent MVD were operated with a standard posterior suboccipital sigmoid sinus approach. Decompression of the trigeminal or facial nerves was performed according to the patient's condition in a free, responsible vessel with the appropriate size of Teflon padding insertion. Simultaneous full exposure of the RVLM, the REZ of the IX and X cranial nerves, and the insertion of appropriately sized Teflon-padded cotton were conducted to separate the responsible artery from the beginning of the cerebral nerve roots. For large or complex vascular compression, multiple points of padding may be required to ensure that cranial nerve compression was relieved, while fully decompressing RVLM, as shown in Figures 1B, C. Postoperative head CT is shown in Figure 1D.

### 2.3 Follow-up and efficacy determination

Blood pressure values were collected 3 days before surgery as a baseline level and at 4 follow-up visits within 12 months after surgery, including 3 days, 3, 6, and 12 months postoperatively. Specific measurements were made by monitoring blood pressure without medication in the morning for three consecutive days before surgery, and the average blood pressure was taken as the preoperative blood pressure. From the 1st day of the postoperative period, the morning blood pressure without medication was monitored for three consecutive days, and the average blood pressure was taken as the 3-day postoperative blood pressure. Postoperative review months (3, 6, and 12 months) were obtained by telephone and clinic visits for three consecutive mornings without blood pressure medication, and the average blood pressure was taken as the blood pressure for each postoperative review month. According to the extent of the decrease in blood pressure and the degree of relief of symptoms, the therapeutic effect was judged as follows: (1) cured: after the patient completely stopped using antihypertensive drugs, systolic blood pressure < 140 mmHg and diastolic blood pressure < 90 mmHg; (2) marked effect: systolic and diastolic blood pressure, one of which decreased to a normal level or systolic blood pressure < 30 mmHg and diastolic blood pressure decreased ≥10 mmHg; (3) effective: systolic blood pressure decreased < 30 mmHg and diastolic blood pressure decreased < 10 mmHg, the clinical symptoms of HTN were relieved, and the dosage of antihypertensive drugs was reduced; and (4) invalidity: the clinical symptoms of HTN were not relieved, and the systolic and diastolic blood pressures were not changed or even increased compared with those before the operation. The marked effect rate and effectiveness rates were calculated for all patients.

Marked effect rate = (cured + marked effect)/total number × 100%.

Effectiveness = (cured + marked effect + effective)/total number × 100%.

By comparing preoperative and postoperative blood pressure levels and assessing the efficacy of MVD, the average reduction in blood pressure or the percentage reduction in systolic/diastolic blood pressure was usually used to measure the change in blood pressure after surgery.

### 2.4 Statistical methods

All data were statistically analyzed using R language software. Normally distributed measures are expressed as the mean ± standard deviation (x ± s). Non-normally distributed data are expressed using a median with an interquartile range. Count data are expressed as frequencies (rates). Continuous data that conformed to the normal distribution were compared using *t*-tests. Continuous data that did not conform to the normal distribution were compared using rank-sum tests, and categorical data were compared using chi-square or Fisher's exact tests. Continuous measurement data, such as blood pressure and heart rate, were compared between the two groups using repeated measures such as Analysis of Variance (ANOVA) or Fridman's test, and two-by-two comparisons were made using Bonferroni or Nemenyi. Comparisons among groups were made using the chi-square test. Differences were considered statistically significant if *p*-values were < 0.05.

## 3 Results

### 3.1 Clinical conditions of patients

A total of 31 male patients and 32 female patients were enrolled, with ages ranging from 39 to 84 years (62.02 ± 10.41), with a history of cranial nerve disease for 6 months to 14 years, and a history of HTN for 1–30 years.

### 3.2 Surgical exploration

In 63 patients, the responsible vessel was VA/BA compression alone in 29 cases (46.03%) or VA/BA complex vascular compression type in 34 cases (53.97%). In the composite vascular compression type, the VA combined with the superior cerebellar artery (10 cases, 29.42%), the anterior inferior cerebellar artery (10 cases, 29.42%), the posterior inferior cerebellar artery (one case, 2.94%), and the petrosal vein (13 cases, 38.24%).

### 3.3 Efficacy analysis

**Figure 2:**
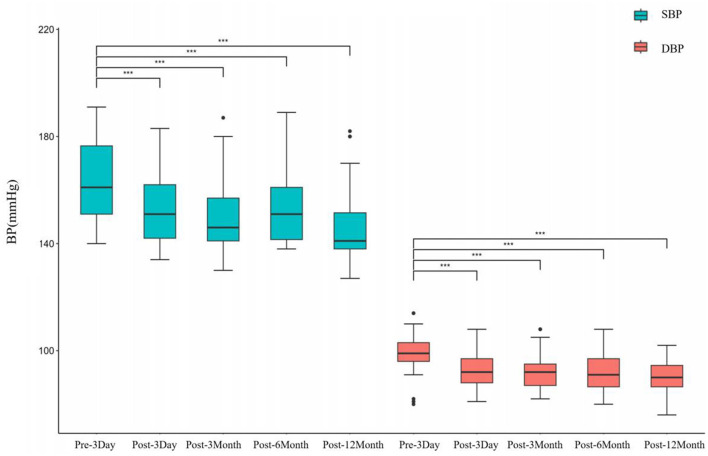
Changes in blood pressure before and after MVD surgery (*** indicates *p* < 0.001).

Briefly, after surgery, patients with a longer history of TN/HFS showed a delayed cure. However, after 1 year of observation and clinical follow-up, clear remissions and the symptoms disappearance occurred among the 63 patients with primary cranial nerve disease. The patients showed a significant decrease in blood pressure after MVD. As shown in Table 1, postoperative systolic and diastolic blood pressure decreased overall throughout the 12-month observation, and the difference was statistically significant. Postoperative blood pressure values showed slight fluctuations, but the difference was not statistically significant. Moreover, the patient's blood pressure reduction was significant and sustained up to 12 months postoperatively, as shown in Figure 2.

**Table 1:** Changes of BP before and after operation in patients (mmHg).

**Time**	**Patients**	
	**SBP**	**DBP**
Pre-3 day	163.94 ± 15.02	99.30 ± 7.51
Post-3 day	154.13 ± 12.87	92.94 ± 6.75
Post-3 month	149.84 ± 13.04	92.41 ± 7.11
Post-6 month	152.27 ± 12.54	91.62 ± 7.26
Post-12 month	146.68 ± 13.49	90.41 ± 6.10
ΔBP	17.25 ± 12.66	8.89 ± 6.39

ΔBP, Changes of blood pressure before and after operation.

According to the postoperative blood pressure, nine cases (14.28%) were cured, eight cases (12.70%) were shown to have a marked effect, 16 cases (25.40%) were effective, and 30 cases (47.62%) were invalid, resulting in an overall efficacy rate of 26.98% and an overall effectiveness rate of 52.38%. Among the 39 cases on the left side, eight cases were cured, eight cases showed a marked effect, and 10 cases were effective, whereas on the right side, one case was cured, and six cases were effective out of 24 cases. The postoperative efficiency was higher on the left side (66.67 > 29.17%) and the difference was statistically significant (*p* < 0.01). We found that patients with a shorter history of cranial nerve disease had a higher postoperative efficiency (70.37 > 38.39%). The difference was similarly statistically significant (*p* < 0.05). However, there was little difference in postoperative efficiency between patients of different genders, different primary cranial nerve disorders, and different responsible vessels. Compared to other subgroups, greater postoperative efficiency was shown in patients younger than 65 years of age, hypertensive class III, and those with a short history of neurogenic HTN. However, the difference was not statistically significant. There was a significant postoperative decrease in blood pressure in patients of both sexes, and a sustained antihypertensive effect was present in all of them. Systolic blood pressure reduction after MVD was greater in patients younger than 65 years than in patients ≥65 years, involving primary TN and left-sided patients. The postoperative decrease in blood pressure after MVD was greater, more predominantly in systolic blood pressure. However, patients with the VA/BA alone as the responsible vessel had higher preoperative blood pressure levels than patients with the VA/BA composite-vessel type, who showed a decrease in systolic blood pressure. The higher the HTN classification and the longer the history of the patient, the greater the decrease in blood pressure after MVD, as shown in Table 2.

**Table 2:** Demographic characteristics and compressive vessels at surgery in the groups of patients.

	**Cases**	**Invalidity**	**Effective**	**Marked effect**	**Cure**	**Effectiveness (%)**	***P*-value**
**Gender**							**0.38**
Male	31	17	5	3	6	45.16	
Female	32	13	11	5	3	54.84	
**Age (year)**							**0.24**
< 65 year	29	11	9	2	7	62.07	
≥65 year	34	19	7	6	2	44.12	
**Combined cranial nerve disease**							**0.67**
HFS	28	12	7	3	6	57.14	
TN	35	18	9	5	3	48.57	
**Affected side**							<**0.01**
Left	39	13	10	8	8	66.67	
Right	24	17	6	0	1	29.17	
**Course of cranial nerve disease**							**0.03**
< 3 years	27	8	11	2	6	70.37	
≥3 years	36	22	5	6	3	38.89	
**Responsible vascularization**							**0.99**
Vertebral/basilar artery	29	14	8	3	4	51.72	
Vertebral/basilar artery complex vascular	34	16	8	5	5	52.94	
**Classification of HTN**							**0.41**
Level I	25	13	5	2	5	48	
Level II	16	9	2	4	1	43.75	
Level III	22	8	9	2	3	63.64	
**Course of HTN (year)**							**0.39**
< 7 years	34	14	10	4	6	58.82	
≥7 years	29	16	6	4	3	44.83	

### 3.4 Surgical complications

No patients developed any serious complications. One patient developed hoarseness and recovered 1 month after surgery; two patients developed postoperative facial numbness and recovered within 2 weeks after surgery; one patient developed facial paralysis 6 days after surgery and fully recovered after 2 months.

### 3.5 Literature review

We reviewed various pieces of literature that were published through 1 December 2023, from the databases PubMed and Embase. The following keywords were searched to find relevant literature and analyses: “ventrolateral medulla,” “RVLM,” “root-entry zone,” “root-entry/exit zone,” “neurogenic,” “medulla oblongata,” “glossopharyngeal nerve,” “nucleus tractus solitaril,” “compression,” “microvascular decompression,” “neurogenic HTN,” and “blood pressure.” A review of seven publications that met the search requirements revealed that the postoperative efficacy of MVD in the treatment of neurogenic HTN varied, with effective rates ranging from as high as 87.5% to as low as 23.8%, as shown in Table 3.

**Table 3:** Cases report of MVD for neurogenic HTN.

**References**	**Cases**	**Average age**	**Combined cranial nerve disease**	**Male/ female**	**Left/ right**	**Therapeutic effect**
Jannetta et al. (13)	42	57.3	Yes	Not mentioned	42/0	Cured 13, significantly effective 18, and effective four cases
Yamamoto et al. (27)	21	53	Yes	15/6	16/5	Effective four cases
Geiger et al. (15)	8	45	No	7/1	Not mentioned	After 3 months, there were three cases of apparent effect and four cases of cure.
Levy et al. (23)	12	52.3	No	5/7	12/0	There were 10 cases of 20 mmHg reduction in systolic blood pressure and five sustained improvements
Frank et al. (17)	8	44	No	1/7	Not mentioned	There were five cases with normal blood pressure within 1 year, three within 2 years
Frank et al. (28)	14	46	No	4/10	14/0	Blood pressure decreases within 1 year, and after 2 years elevated to preoperative levels
Legrady et al. (24)	13	44	No	3/10	13/0	Significant decrease in blood pressure in 13 cases
Sindou et al. (18)	48	60	Yes	22/26	30/18	Cured 14, significantly effective 14, effective 10 cases
Lu et al. (26)	30	60.6	Yes	17/13	30/0	Cured 10, significantly effective nine, and effective six cases

## 4 Discussion

Alexander et al. in 1946 found that stimulation of the RVLM resulted in increased blood pressure. It is now widely accepted that the vasodilatory center is located in the RVLM (16). The ventral lateral medulla contains C1 adrenergic cells that release fibers that project to the mediolateral columns of the spinal cord. Fibers then regulate cardiovascular activity through sympathetic innervation of the adrenal medulla, heart, and blood vessels, triggering sympathetic arousal, and eventually the blood pressure rises. Regarding the etiology of neurogenic HTN, in 1973, Jannetta et al. reported for the first time that a patient with neurogenic HTN underwent a successful MVD. It resulted in effective control of the patient's blood pressure. This finding lays the foundation for further research on neurogenic HTN (4). Between 1975 and 1982, Jannetta et al. performed MVD in 42 patients with neurogenic HTN suffering from marked vascular compression of the left ventral lateral medulla and the REZ of the IX and X cranial nerves. The results showed that postoperative blood pressure returned to normal in 32 patients and improved in 4 patients, while no significant change was observed in 6 patients. This finding validates the hypothesis from a clinical point of view (4, 13, 14). In 1985, Jannetta et al. (14) also confirmed the idea of vascular compression of the RVLM by pulsatile vessels through animal models, cadaveric dissection, and imaging studies. The efficacy of MVD in the treatment of neurogenic HTN has been further validated. Frank et al. (17) and Sindou et al. (18) have successfully confirmed the efficacy of MVD in the treatment of neurogenic HTN in retrospective studies. Currently, most opinions believe that RVLM is involved in the regulation of blood pressure by participating in the sympathetic system (13, 14, 19). The majority view is that the RVLM mediates blood pressure changes by participating in the sympathetic nerve and renin-angiotensin-aldosterone systems (20, 21). After applying MVD in patients with neurogenic HTN, plasma norepinephrine and urinary epinephrine levels decreased, which also indicated that the HTN of the patients was caused by vascular pulsatile compression of the RVLM stimulating the sympathetic nerves and the renin-angiotensin-aldosterone system, thus causing HTN (22).

Levy et al. (23) implemented MVD in 12 cases of neurogenic HTN, and 10 patients experienced a decrease in systolic blood pressure of more than 20 mmHg. Studies conducted by Legrady et al. (24) explored long-term blood pressure monitoring in patients with neurogenic HTN before and after MVD surgery. The results of the study showed a significant decrease in the patient's blood pressure. In our study, the MVD was performed on 63 patients with a preoperative diagnosis of neurogenic HTN, and the result showed an overall effectiveness rate of 52.38%. Among the 39 cases on the left side, eight cases were cured, eight cases showed obvious effects, and 10 cases were effective; among the 24 cases on the right side, one case was cured, and six cases were effective. This is consistent with previous findings. The efficiency rate of the left side was 66.67%, which was statistically significantly higher, while the efficiency rate of the right side was 29.16%. This suggests that the left RVLM and the REZ of the IX and X cranial nerves play a very important role in blood pressure regulation. Although many experts and scholars suggest that the cause of neurogenic HTN is vascular compression in the left RVLM and the REZ of the IX and X cranial nerves, these studies failed to explain why the medulla oblongata has a left-sided advantage in cardiovascular regulation. One possible explanation is that myocardial receptor afferent impulses from the left ventricle and atria are conducted to the nucleus of the solitary tract primarily through cardiac c-fibers of the left vagus nerve. However, mechanical damage to the nerve by blood vessels may block this conduction, leading to a decrease in afferent impulses received by the nucleus tractus solitarius and, finally, to the development of HTN. It has also been shown that there is no significant difference in the efficacy of RVLM areas on the left and right sides after MVD surgery (25). However, some studies show that the left and right sides do not play a role in the regulation of blood pressure, thus further validation is still needed (26).

Furthermore, postoperative outcomes vary. Among them, Geiger et al. (15) showed an effective rate of 87.5% after MVD treatment in patients with neurogenic HTN. In contrast, a study by Yamamoto et al. (27) showed that the effective rate was only 23.8%. The results of this study showed that the overall effectiveness of MVD in the treatment of neurogenic HTN was 52.38%. The overall postoperative blood pressure profile was slightly lower than in previous studies (18). However, postoperative blood pressure did not improve in some of the patients in this trial; this may be due to differences in the individual circumstances of different patients. These patients may be associated with chronic vascular compression of the vagus nerve, resulting in an inability to recover immediately after decompression (23, 24). Meanwhile, through clinical observation, we conjecture that TN/HFS can also aggravate HTN, which is mainly due to prolonged and repeated pain stimulation, tension anxiety, and sympathetic excitation. MVD reduces pain and anxiety symptoms. Therefore, given the presence of abnormal blood pressure compressions in the RVLM and REZ regions, it has been suggested that repeated and prolonged pain and anxiety may tend to cause elevated blood pressure (22, 26). It is possibly related to the number of cases, after which it still needs to be validated in a large sample trial. In addition, the duration of blood pressure follow-up also affects the efficiency of MVD (17).

The difference between our study and other studies (18, 24, 28) is that we selected vertebrobasilar TN/HFS for MVD. The VA/BA type requires multipoint spacers, and intraoperative use of padded cotton spacers in the RVLM and the REZ of the IX and X cranial nerves is routinely required. The surgery provides adequate decompression to ensure the resolution of cranial nerve disease and also ensures effectiveness in addressing primary cranial nerve disorders. Other responsible vessel types of TN/HFS require additional intraoperative exploration of the RVLM, which again increases the surgical risk to the patient. MVD may have different levels of efficacies in different patients, and this study focused on the analysis of patients with VA/BA type cranial nerve disease combined with neurogenic HTN. This is because the dilatation and positional variations of the vertebral arteries tend to involve both the cranial nerves and the ventral lateral medulla oblongata. However, VA variations are most commonly seen in VA/BA dolichoectasia (29, 30). VBD is a condition in which the vertebral or basilar arteries are abnormally dilated and lengthened due to abnormalities in the walls of the blood vessels caused by a number of hereditary or autoimmune diseases and other factors (31, 32). Therefore, the VA/BA with anatomical variants of the vessel and an abnormal location is more likely to cause compression of the medulla oblongata and posterior group of cranial nerves in the REZ region in the context of the narrow space of the posterior cranial fossa. The VA/BA has a thicker diameter, leading to a greater compressive force, which is also responsible for causing neurogenic HTN (33). We also collected 106 cases of HTN combined with TN/HFS in other responsible vessel types. We performed MVD of the V/VII cranial nerve during surgery to address the patient's primary cranial nerve disease. We observed a poor reduction in blood pressure after MVD compared to preoperative blood pressure; the change in systolic blood pressure was 7.61 ± 9.96 mmHg and diastolic blood pressure was 5.05 ± 7.76 mmHg. Therefore, we suggest that pain and anxiety could be one of the reasons for the drop in blood pressure. However, due to the low drop in blood pressure, these factors could not be the underlying cause.

MVD treats neurogenic HTN by appropriate decompression of the RVLM along with the cranial nerve disease. In addition, the difference in efficacy after surgery and instability related to the adequacy of decompression still needs to be validated with more surgical cases in a prospective multicenter study.

Since many patients with neurogenic HTN are not fully amenable to surgical treatment, the indications for surgery for neurogenic HTN and the long-term outcome remain unclear. The direct treatment of neurogenic HTN with MVD is not commonly recognized internationally. The cases selected for our central study also had comorbid cranial nerve disorders. Primarily due to the same surgical access, the RVLM was incidentally explored and decompressed while the VA/BA compression was addressed intraoperatively. Indications for surgery may include the following: left-sided TN or HFS combined with neurogenic HTN; a cranial MRI showed VA/BA compression in the left RVLM and the REZ region of the IX and X cranial nerves; and blood pressure cannot be effectively controlled by medication. Care should be taken with neurogenic HTN patients who are difficult to control medically and whose imaging confirms vascular compression of the RVLM and the REZ of the IX and X cranial nerves by implementing MVD for these two regions. The beneficiary group is likely to be much higher than the status quo.

In the future, with the development and advancement of pathophysiology and imaging techniques, physicians will have a deeper understanding of the pathogenesis, clinical features, and imaging of neurogenic HTN. Meanwhile, surgical understanding is improving, allowing for better treatment of patients with neurogenic HTN. MVD is an effective treatment for neurogenic HTN. Compared with previous research studies, we have taken a further step in the theoretical and practical aspects of MVD with a larger sample size and more systematic analysis, which provides the possibility for further exploration of this field in the future.

## 5 Conclusion

MVD is an effective treatment for neurogenic HTN. However, the criteria for selecting patients who need MVD to control their HTN still need to be further defined. Indications for MVD may include as follows: left-sided TN or HFS combined with neurogenic HTN; VA/BA compression in the left RVLM and REZ areas on MRI; and blood pressure in these patients which cannot be effectively controlled by drugs.

## Data availability statement

The raw data supporting the conclusions of this article will be made available by the authors, without undue reservation.

## Ethics statement

The studies involving humans were approved by Ethics Committee of The First Affiliated Hospital of Dalian Medical University. The studies were conducted in accordance with the local legislation and institutional requirements. Written informed consent for participation was not required from the participants or the participants' legal guardians/next of kin in accordance with the national legislation and institutional requirements.

## Author contributions

DZ: Conceptualization, Data curation, Supervision, Validation, Writing – original draft, Writing – review & editing. SW: Writing – review & editing, Formal analysis, Investigation, Methodology, Writing – original draft. XW: Formal analysis, Investigation, Methodology, Writing – review & editing. XH: Data curation, Writing – review & editing. SZ: Conceptualization, Data curation, Writing – review & editing. HZ: Data curation, Software, Writing – review & editing. XF: Formal analysis, Project administration, Validation, Writing – review & editing. YL: Formal analysis, Project administration, Validation, Writing – review & editing. ZW: Conceptualization, Data curation, Formal analysis, Funding acquisition, Investigation, Methodology, Project administration, Resources, Software, Supervision, Validation, Visualization, Writing – original draft, Writing – review & editing.

## References

[1] WHO. Hypertension. (2023). Available online at: https://www.who.int/news-room/fact-sheets/detail/hypertension (accessed March 16, 2023).

[2] NCD Risk Factor Collaboration. Worldwide trends in hypertension prevalence and progress in treatment and control from 1990 to 2019, a pooled analysis of 1201 population-representative studies with 104 million participants. Lancet. (2021) 398:957–80. 10.1016/S0140-6736(21)01330-1 34450083 PMC8446938

[3] Hering D Kara T Kucharska W Somers VK Narkiewicz K. High-normal blood pressure is associated with increased resting sympathetic activity but normal responses to stress tests. Blood Press. (2013) 22:183–7. 10.3109/08037051.2012.759689 23356493 PMC3951917

[4] Jannetta PJ Gendell HM. Clinical observations on etiology of essential hypertension. Surg Forum. (1979) 30:431–2. 538657

[5] Oyarce MP Iturriaga R. Contribution of oxidative stress and inflammation to the neurogenic hypertension induced by intermittent hypoxia. Front Physiol. (2018) 9:893. 10.3389/fphys.2018.00893 30050461 PMC6050421

[6] Mann SJ. Neurogenic hypertension: pathophysiology, diagnosis and management. Clin Autonom Res. (2018) 28:363–74. 10.1007/s10286-018-0541-z 29974290

[7] Sakuma T Morimoto S Aota Y Takahashi N Toyoda N Kosaki A. Efficacy of clonidine in patients with essential hypertension with neurovascular contact of the rostral ventrolateral medulla. Hypert. Res. (2010) 33:633–7. 10.1038/hr.2010.41 20379192

[8] Kleineberg B Becker H Gaab MR Naraghi R. Essential hypertension associated with neurovascular compression: angiographic findings. Neurosurgery. (1991) 30:834–41. 10.1227/00006123-199206000-00003 1614583

[9] Naraghi R Gaab MR Walter GF Kleineberg B. Arterial hypertension and neurovascular compression at the ventrolateral medulla. A comparative microanatomical and pathological study. J Neurosurg. (1992) 77:103–12. 10.3171/jns.1992.77.1.0103 1307855

[10] Akimura T Furutani Y Jimi Y Saito K Kashiwagi S Kato S. Essential hypertension and neurovascular compression at the ventrolateral medulla oblongata: MR evaluation. Am J Neuroradiol. (1995) 16:401–5. 7726090 PMC8338326

[11] Morimoto S Sasaki S Miki S Kawa T Itoh H Nakata T. Pulsatile compression of the rostral ventrolateral medulla in hypertension. Hypertension. (1997) 29:514–8. 10.1161/01.HYP.29.1.514 9039152

[12] Morimoto S Sasaki S Miki S Kawa T Itoh H Nakata T. Neurovascular compression of the rostral ventrolateral medulla related to essential hypertension. Hypertension. (1997) 30:77–82. 10.1161/01.HYP.30.1.77 9231824

[13] Jannetta PJ Segal R Wolfson SKJr. Neurogenic hypertension: etiology and surgical treatment. I. Observations in 53 patients. Ann Surg. (1985) 201:391–8. 10.1097/00000658-198503000-00023 3977442 PMC1250685

[14] Jannetta PJ Segal R Wolfson SKJr Dujovny M Semba A Cook EE. Neurogenic hypertension: etiology and surgical treatment. II. Observations in an experimental nonhuman primate model. Ann Surg. (1985) 202:253–61. 10.1097/00000658-198508000-00018 4015232 PMC1250882

[15] Geiger H Naraghi R Schobel HP Frank H Sterzel RB Fahlbusch R. Decrease of blood pressure by ventrolateral medullary decompression in essential hypertension. Lancet. (1998) 352:446–9. 10.1016/S0140-6736(97)11343-5 9708753

[16] Alexander RS. Tonic and reflex functions of medullary sympathetic cardiovascular centers. J Neurophysiol. (1946) 9:205–17. 10.1152/jn.1946.9.3.205 21028163

[17] Frank H Schobel HP Heusser K Geiger H Fahlbusch R Naraghi R. Long-term results after microvascular decompression in essential hypertension. Stroke. (2001) 32:2950–5. 10.1161/hs1201.099799 11740004

[18] Sindou M Mahmoudi M Brînzeu A. Hypertension of neurogenic origin: effect of microvascular decompression of the CN IX-X root entry/exit zone and ventrolateral medulla on blood pressure in a prospective series of 48 patients with hemifacial spasm associated with essential hypertension. J Neurosurg. (2015) 123:1405–13. 10.3171/2014.12.JNS141775 26230479

[19] Fein JM Frishman W. Neurogenic hypertension related to vascular compression of the lateral medulla. Neurosurgery. (1980) 6:615–22. 10.1227/00006123-198006000-00001 7432603

[20] Barley J Ellis C. Microvascular decompression surgery for refractory hypertension of neurogenic causes. J Clin Hypert. (2013) 15:217. 10.1111/jch.12019 23458596 PMC8033799

[21] Zhu J Gu R Ji F. Microvascular decompression can effectively reduce arterial blood pressure in patients with Trigeminal Neuralgia. Clin Neurol Neurosurg. (2023) 233:107945. 10.1016/j.clineuro.2023.107945 37611352

[22] Sasaki S Tanda S Hatta T Morimoto S Takeda K Kizu O. Neurovascular decompression of the rostral ventrolateral medulla decreases blood pressure and sympathetic nerve activity in patients with refractory hypertension. J Clin Hypert. (2011) 13:818–20. 10.1111/j.1751-7176.2011.00522.x 22051426 PMC8108941

[23] Levy EI Clyde B McLaughlin MR Jannetta PJ. Microvascular decompression of the left lateral medulla oblongata for severe refractory neurogenic hypertension. Neurosurgery. (1998) 43:1–6. 10.1097/00006123-199807000-00001 9657182

[24] Legrady P Voros E Bajcsi D Fejes I Barzo P Abraham G. Observations of changes of blood pressure before and after neurosurgical decompression in hypertensive patients with different types of neurovascular compression of brain stem. Kidney Blood Press Res. (2013) 37:451–7. 10.1159/000355725 24247558

[25] Thorén P. Role of cardiac vagal C-fibers in cardiovascular control. Rev Physiol Biochem Pharmacol. (1979) 86:1–94. 10.1007/BFb0031531 386467

[26] Lu W Wang H Yan Z Wang Y Che H. Microvascular decompression for the treatment of neurogenic hypertensi on with trigeminal neuralgia. BMC Neurol. (2019) 19:341. 10.1186/s12883-019-1569-y 31881866 PMC6933738

[27] Yamamoto I Yamada S Sato O. Microvascular decompression for hypertension–clinical and experimental study. Neurol Med Chir. (1991) 31:1–6. 10.2176/nmc.31.1 1712916

[28] Frank H Heusser K Geiger H Fahlbusch R Naraghi R Schobel HP. Temporary reduction of blood pressure and sympathetic nerve activity in hypertensive patients after microvascular decompression. Stroke. (2009) 40:47–51. 10.1161/STROKEAHA.108.518670 18927455

[29] Zheng W Wang L Wang H Zhou H Du Q. Trigeminal neuralgia caused by vertebrobasilar dolichoectasia: efficacy of stepwise decompression technique. Acta Neurochir. (2023) 165:3019–26. 10.1007/s00701-023-05691-7 37353618

[30] El-Ghandour NMF. Microvascular decompression in the treatment of trigeminal neuralgia caused by vertebrobasilar ectasia. Neurosurgery. (2010) 67:330–7. 10.1227/01.NEU.0000371978.86528.60 20644418

[31] Dziewasa R Freund M Lüdemann P Müller M Ritter M Droste DW. Treatment options in vertebrobasilar dolichoectasia–case report and review of the literature. Eur Neurol. (2003) 49:245–7. 10.1159/000070196 12736544

[32] Wolters FJ Rinkel GJ Vergouwen MD. Clinical course and treatment of vertebrobasilar dolichoectasia: a systematic review of the literature. Neurol Res. (2013) 35:131–7. 10.1179/1743132812Y.0000000149 23452575

[33] Pico F Labreuche J Amarenco P. Pathophysiology, presentation, prognosis, and management of intracranial arterial dolichoectasia. Lancet Neurol. (2015) 14:833–45. 10.1016/S1474-4422(15)00089-7 26194931

