# Supplementary material for: The effect of microvascular decompression of the CN IX-X root entry/exit zone and the ventrolateral medulla in neurogenic hypertension involving the vertebral/basilar artery
Source: Front Neurol. 2024 Jun 18;15:1376019. doi: 10.3389/fneur.2024.1376019 (PMC11218759; doi:10.3389/fneur.2024.1376019)
Supplement: Supplementary file 1 [file Data_Sheet_1.docx]

Supplementary Material

**Effect of microvascular decompression of CN IX-X root entry/exit zone and ventrolateral medulla for neurogenic hypertension involving the vertebral/basilar artery**

## 1.Supplementary Figures

**Supplementary Figure 1.** Subgroup analysis-Male

**Supplementary Figure 2.** Subgroup analysis-Female

**Supplementary Figure 3.** Subgroup analysis-Age<65years

**Supplementary Figure 4.** Subgroup analysis-Age≥65years

**Supplementary Figure 5.** Subgroup analysis-HFS

**Supplementary Figure 6.** Subgroup analysis-TN

**Supplementary Figure 7.** Subgroup analysis-Left

**Supplementary Figure 8.** Subgroup analysis-Right

**Supplementary Figure 9.** Subgroup analysis-Course of cranial nerve disease < 3 years

**Supplementary Figure 10.** Subgroup analysis-Course of cranial nerve disease ≥ 3 years

**Supplementary Figure 11.** Subgroup analysis-Vetebral / basilar artery

**Supplementary Figure 12.** Subgroup analysis-Vetebral / basilar artery complex vascular

**Supplementary Figure 13.** Subgroup analysis-HTN Level I

**Supplementary Figure 14.** Subgroup analysis-HTN Level Ⅱ

**Supplementary Figure 15.** Subgroup analysis-HTN Level Ⅲ

**Supplementary Figure 16.** Subgroup analysis-Course of HTN ＜ 7 years

**Supplementary Figure 17.** Subgroup analysis-Course of HTN ≥7 years

**Supplementary Table 1.** The medication of 33 patients with effective hypertension after MVD


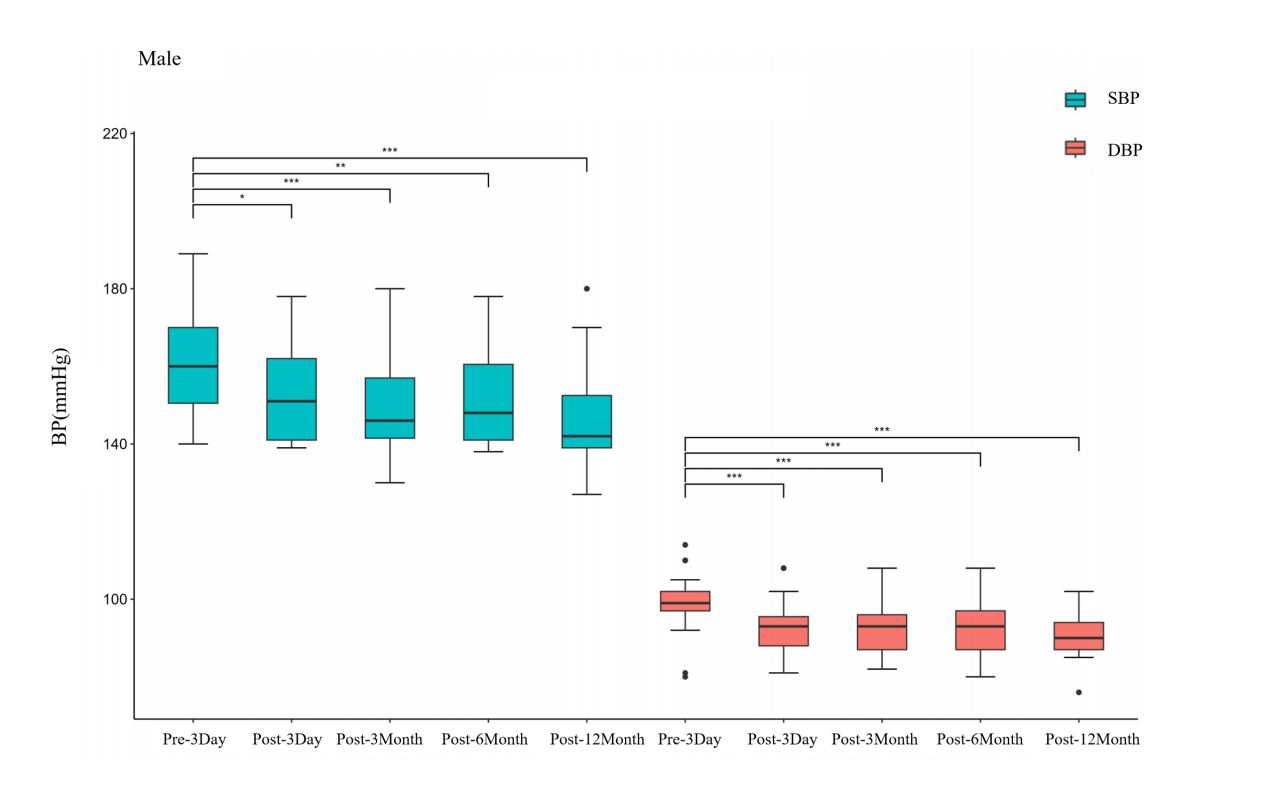


**Supplementary Figure 1.** Subgroup analysis-Male ：There was no significant difference in preoperative blood pressure levels between patients of different genders, and postoperative blood pressure decreased significantly (P<0.05), and there was a sustained antihypertensive effect in all of them, and no significant difference in blood pressure changes between men and women was found, as shown in Figures 1 and 2.


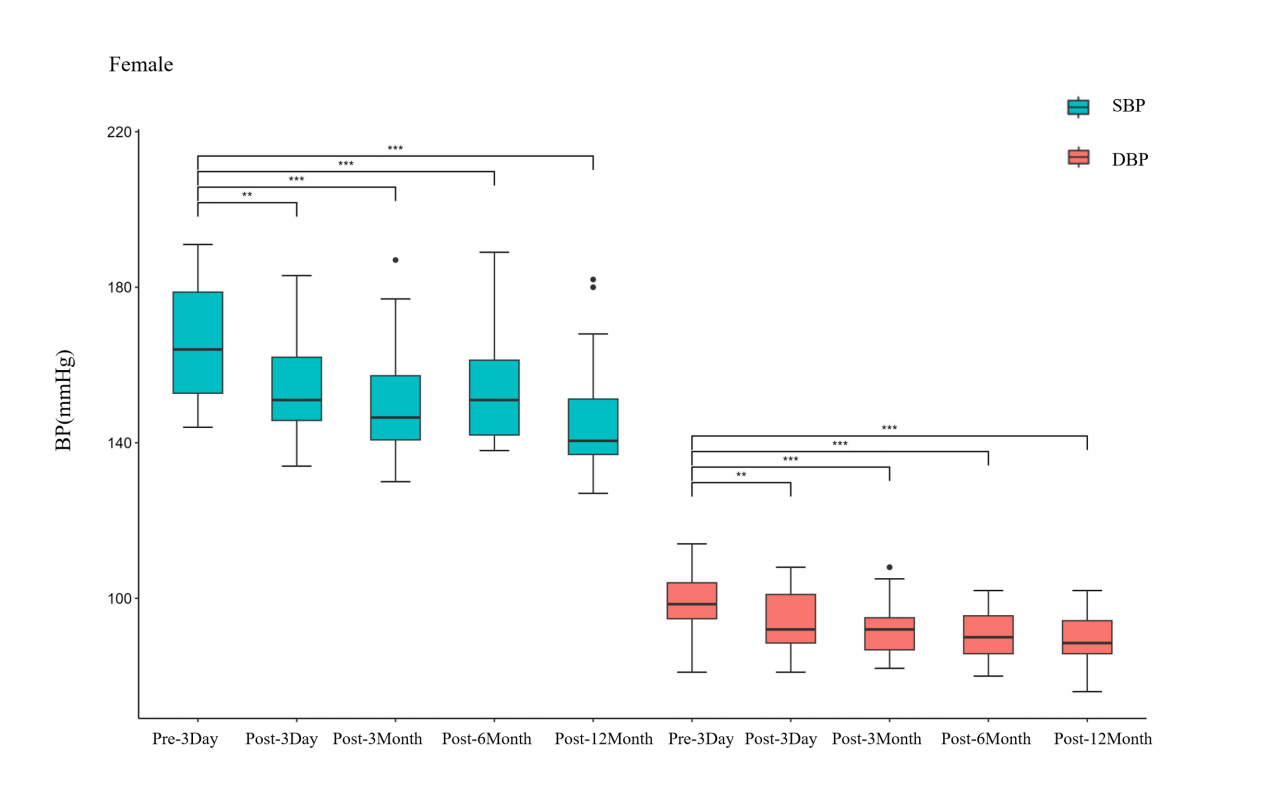


**Supplementary Figure 2.** Subgroup analysis-Female：There was no significant difference in preoperative blood pressure levels between patients of different genders, and postoperative blood pressure decreased significantly (P<0.05), and there was a sustained antihypertensive effect in all of them, and no significant difference in blood pressure changes between men and women was found, as shown in Figures 1 and 2.


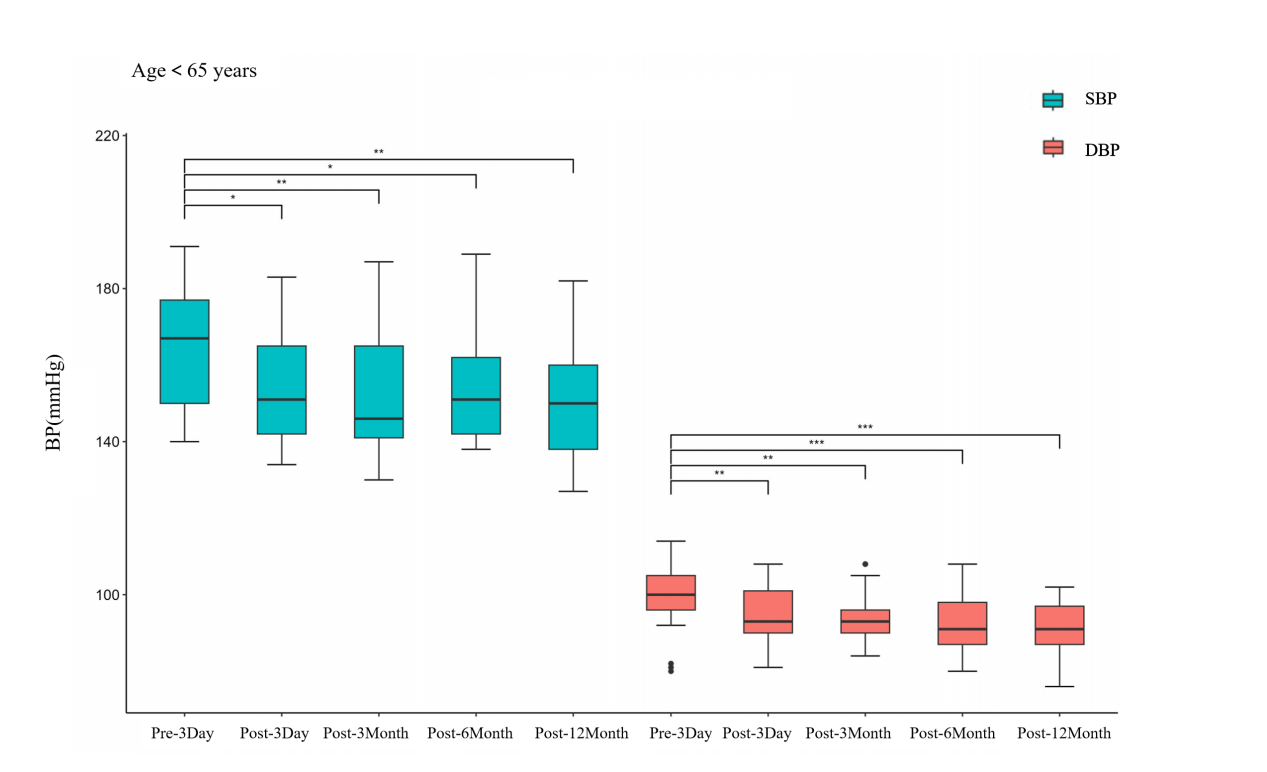


**Supplementary Figure 3.** Subgroup analysis-Age<65years：Blood pressure decreased significantly after MVD in patients of different ages (P<0.05), and systolic blood pressure decreased by approximately 18 mmHg after surgery in patients <65 years old, which was greater than that in patients ≥65 years old (9 mmHg), and there was a sustained blood pressure lowering effect in both, as shown in Figures 3 and 4.


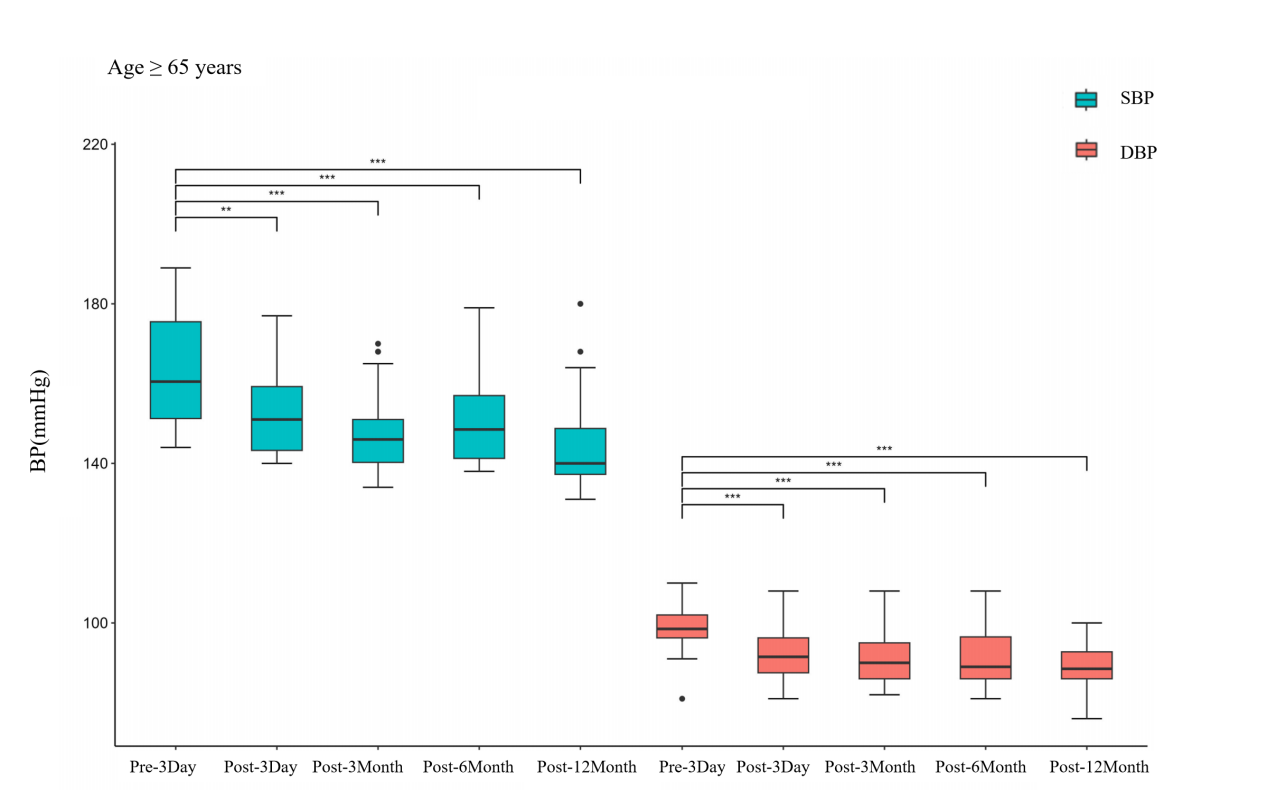


**Supplementary Figure 4.** Subgroup analysis-Age≥65years：Blood pressure decreased significantly after MVD in patients of different ages (P<0.05), and systolic blood pressure decreased by approximately 18 mmHg after surgery in patients <65 years old, which was greater than that in patients ≥65 years old (9 mmHg), and there was a sustained blood pressure lowering effect in both, as shown in Figures 3 and 4.


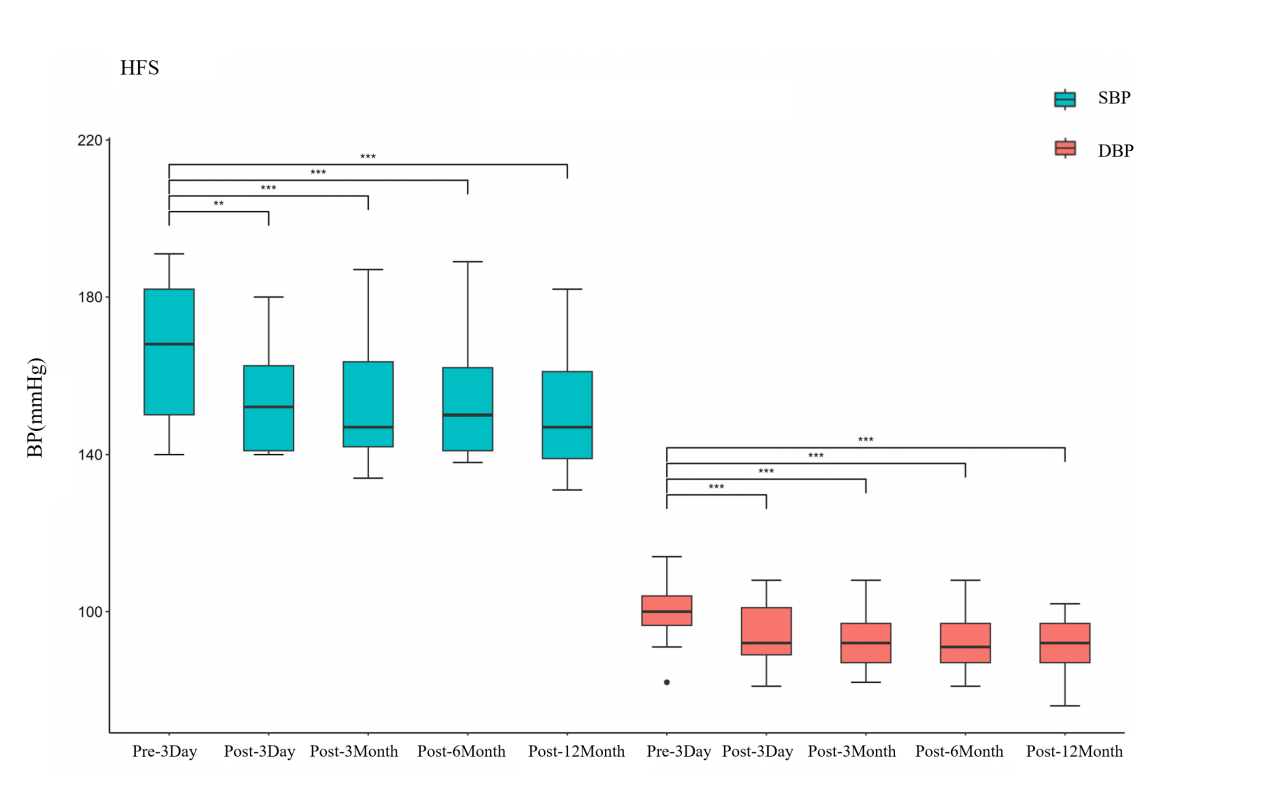


**Supplementary Figure 5.** Subgroup analysis-HFS：Preoperative blood pressure levels were higher in patients with trigeminal nerve involvement than in patients with facial nerve involvement, and the decrease in systolic blood pressure was greater (approximately 19 mmHg), as detailed in Figures 5 and 6.There was a significant decrease in postoperative blood pressure after MVD in patients with different nerve involvement (P<0.05), and there was a sustained blood pressure lowering effect in all of them.


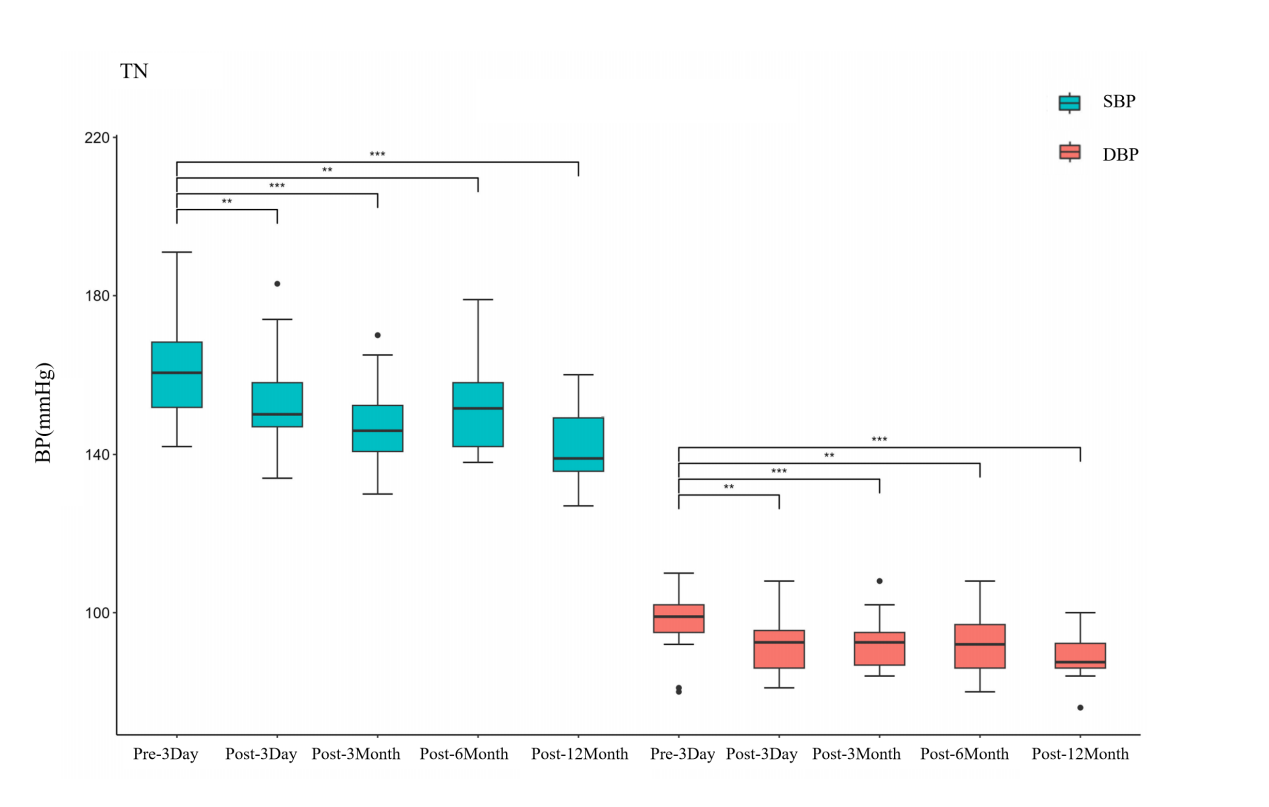


**Supplementary Figure 6.** Subgroup analysis-TN：Preoperative blood pressure levels were higher in patients with trigeminal nerve involvement than in patients with facial nerve involvement, and the decrease in systolic blood pressure was greater (approximately 19 mmHg), as detailed in Figures 5 and 6.There was a significant decrease in postoperative blood pressure after MVD in patients with different nerve involvement (P<0.05), and there was a sustained blood pressure lowering effect in all of them


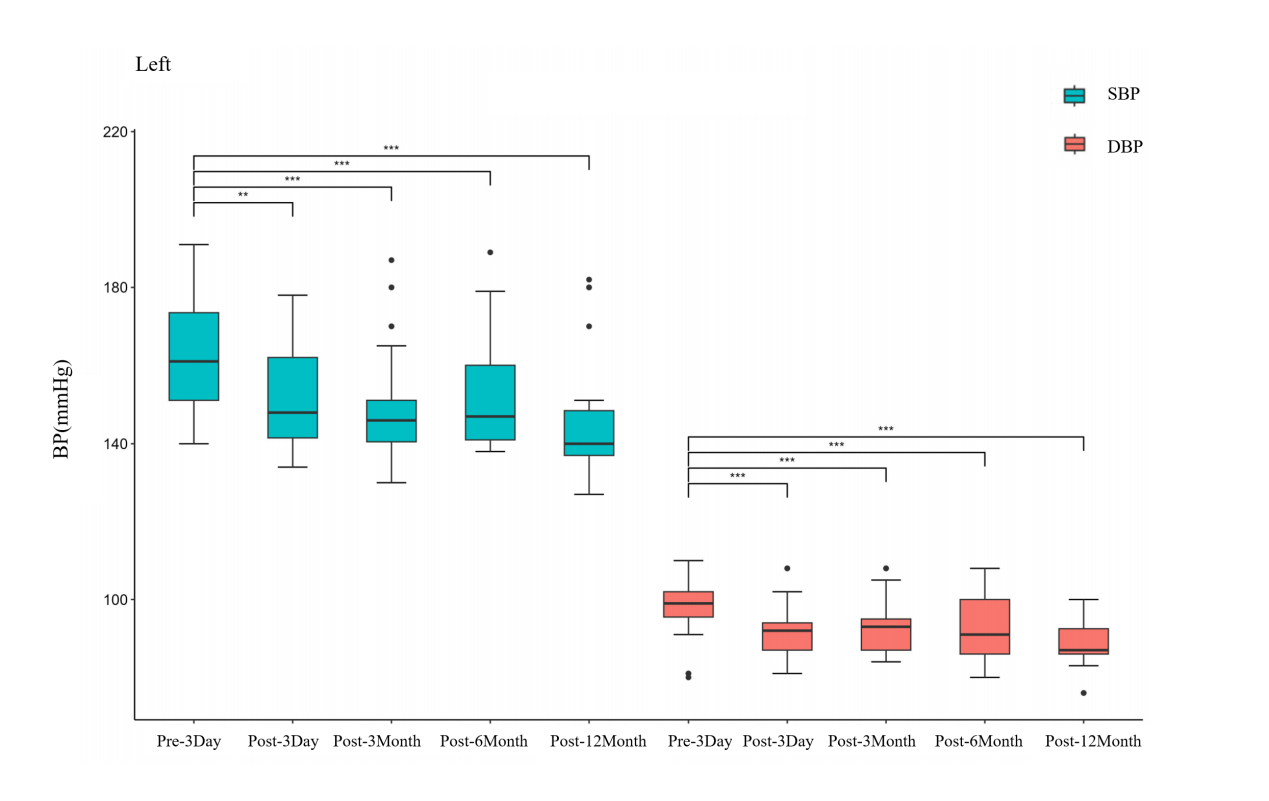


**Supplementary Figure 7.** Subgroup analysis-Left：Compared with the right-sided nerves, patients with involvement of the left-sided nerves had a greater decrease in blood pressure, mainly in systolic blood pressure, as shown in Figures 7 and 8.Patients with involvement of the right-sided nerves did not have a significant effect on the decrease in blood pressure at 3 days after MVD (P>0.05), and their blood pressures were lower than the preoperative levels from 3 to 12 months after surgery (P<0.05).


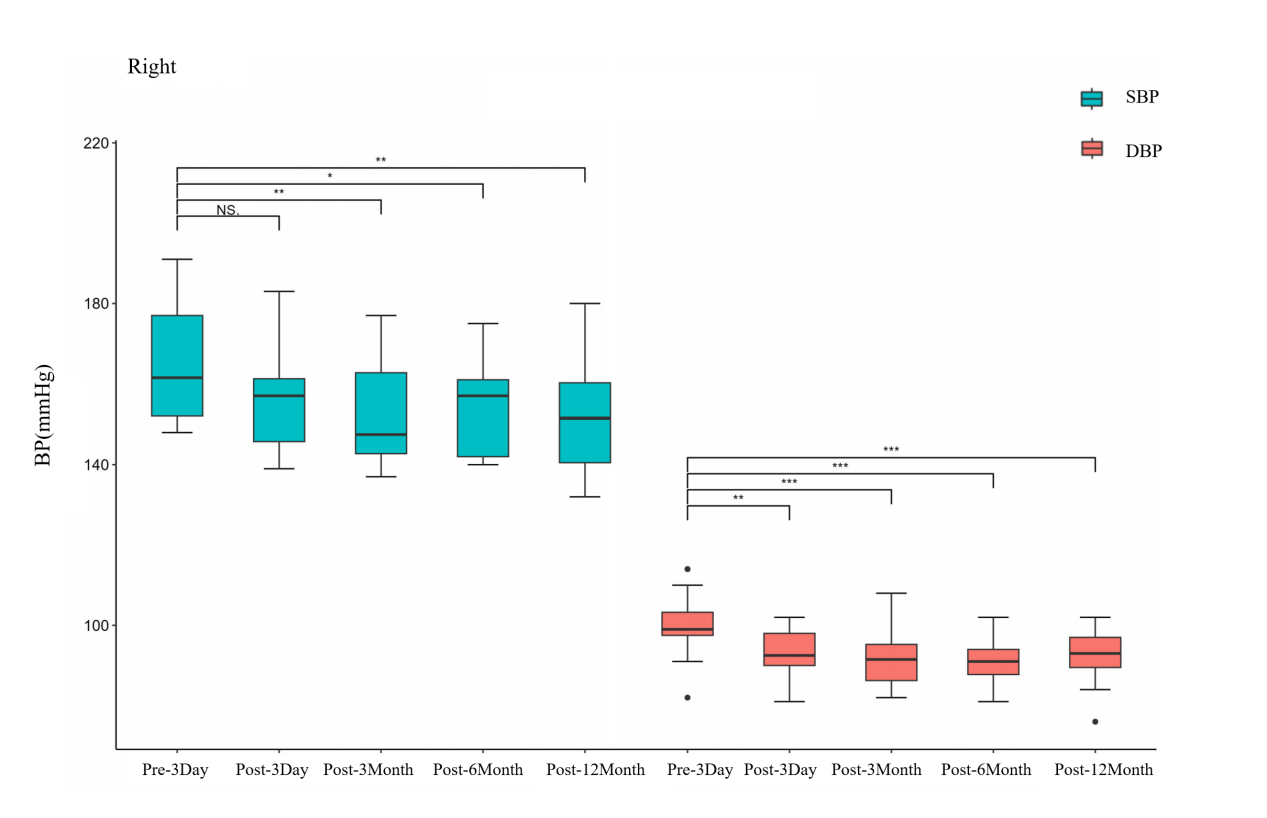


**Supplementary Figure 8.** Subgroup analysis-Right：Compared with the right-sided nerves, patients with involvement of the left-sided nerves had a greater decrease in blood pressure, mainly in systolic blood pressure, as shown in Figures 7 and 8.Patients with involvement of the right-sided nerves did not have a significant effect on the decrease in blood pressure at 3 days after MVD (P>0.05), and their blood pressures were lower than the preoperative levels from 3 to 12 months after surgery (P<0.05).


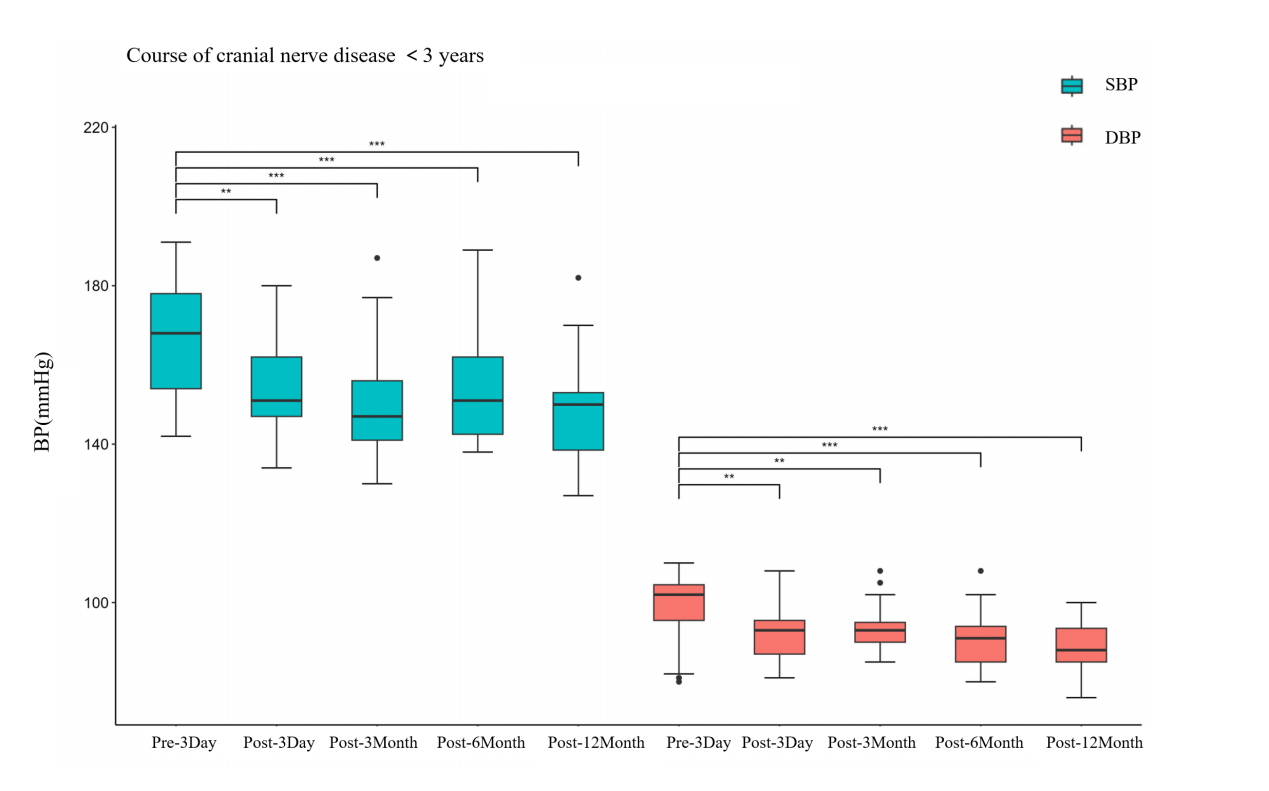


**Supplementary Figure 9.** Subgroup analysis-Course of cranial nerve disease < 3 years：Patients were divided into two groups using the median history of cranial nerve disease (3 years) as the division point. Patients with a shorter history of cranial nerve disease had a better blood pressure reduction than those with a longer history, mainly in systolic blood pressure, as shown in Figures 9 and 10.Patients with different lengths of history of cranial nerve disease had a significant reduction in blood pressure after MVD (P<0.05), and there was a sustained antihypertensive effect in all of them.


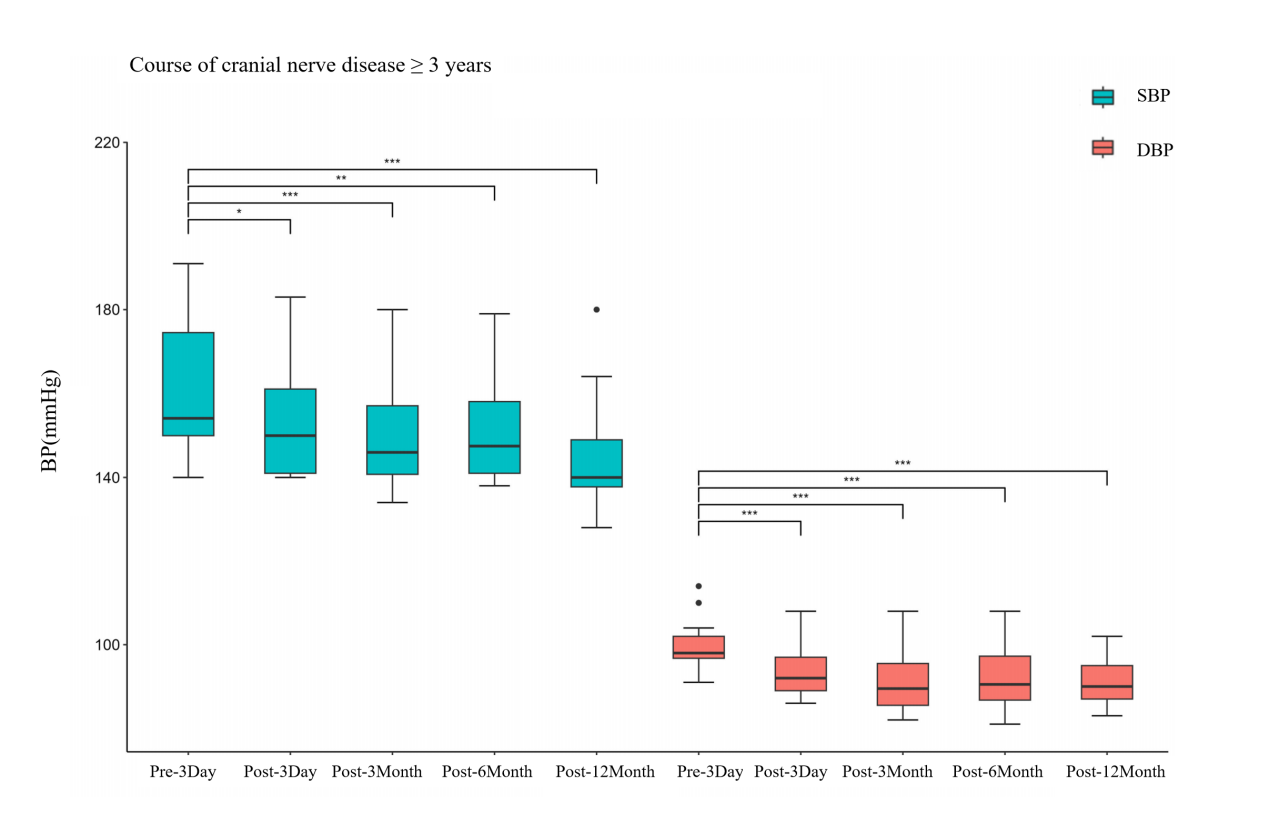


**Supplementary Figure 10.** Subgroup analysis-Course of cranial nerve disease ≥ 3 years：Patients were divided into two groups using the median history of cranial nerve disease (3 years) as the division point. Patients with a shorter history of cranial nerve disease had a better blood pressure reduction than those with a longer history, mainly in systolic blood pressure, as shown in Figures 9 and 10.Patients with different lengths of history of cranial nerve disease had a significant reduction in blood pressure after MVD (P<0.05), and there was a sustained antihypertensive effect in all of them.


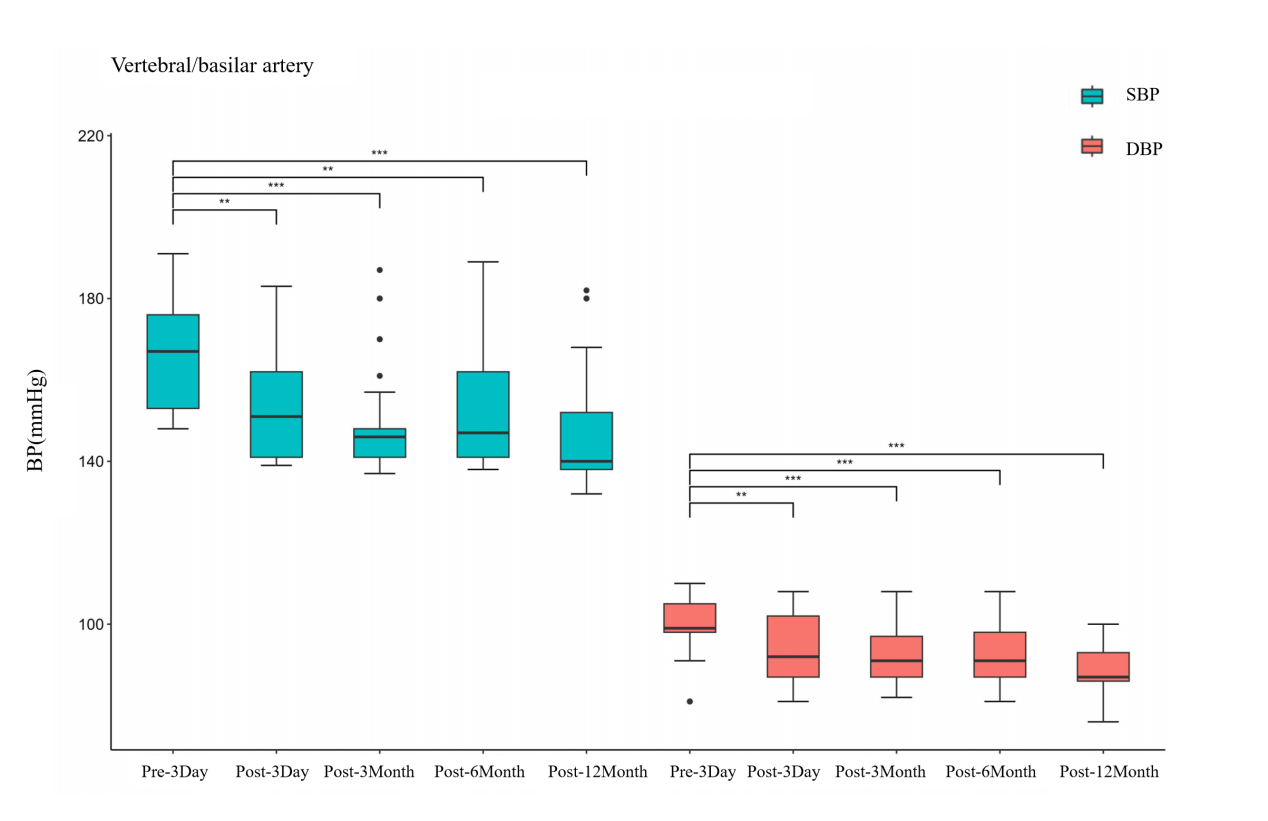


**Supplementary Figure 11.** Subgroup analysis-Vetebral / basilar artery：The preoperative blood pressure levels of patients with vertebral artery alone type were higher than those of patients with composite vessel type, and the decrease in systolic blood pressure was greater, as detailed in Figures 11 and 12.The postoperative decrease in blood pressure after MVD was significant in patients with different responsible vessels (P<0.05), and all of them had sustained blood pressure lowering effect.


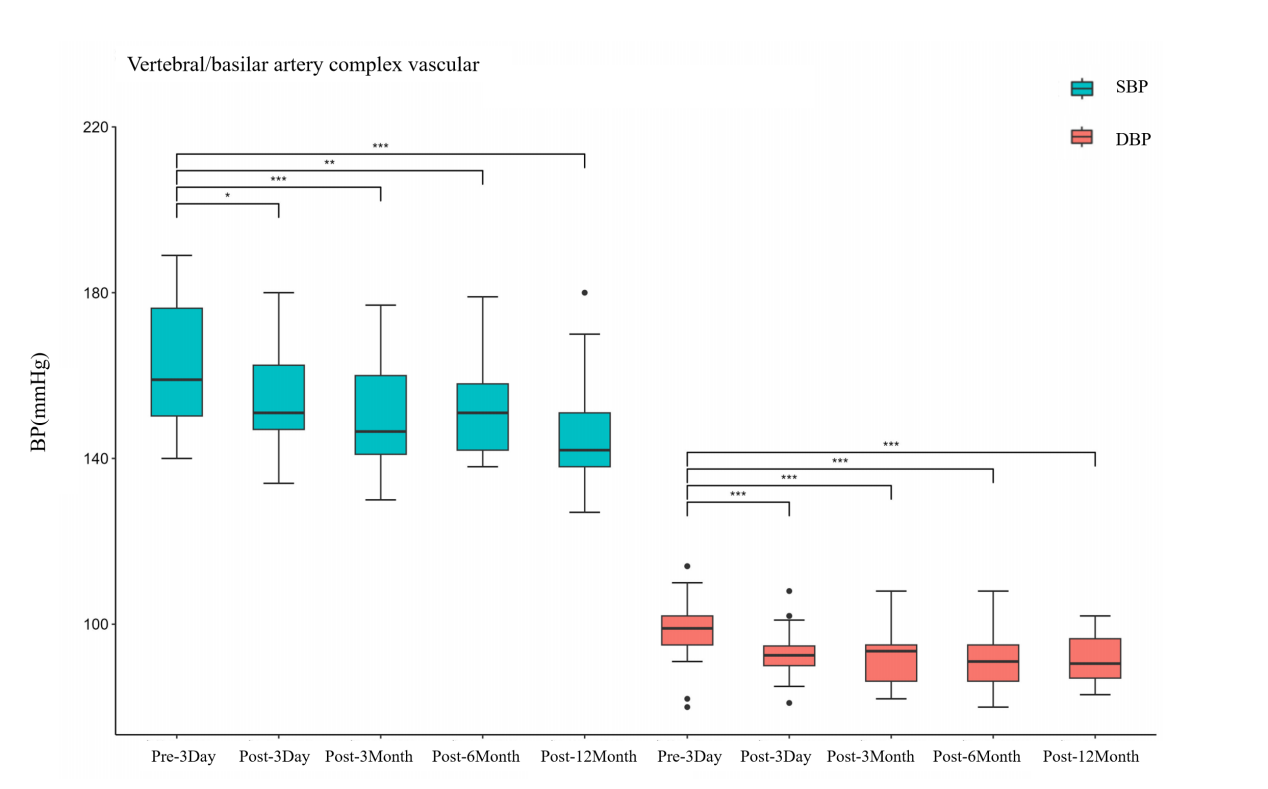


**Supplementary Figure 12.** Subgroup analysis-Vetebral / basilar artery complex vascular：The preoperative blood pressure levels of patients with vertebral artery alone type were higher than those of patients with composite vessel type, and the decrease in systolic blood pressure was greater, as detailed in Figures 11 and 12.The postoperative decrease in blood pressure after MVD was significant in patients with different responsible vessels (P<0.05), and all of them had sustained blood pressure lowering effect.


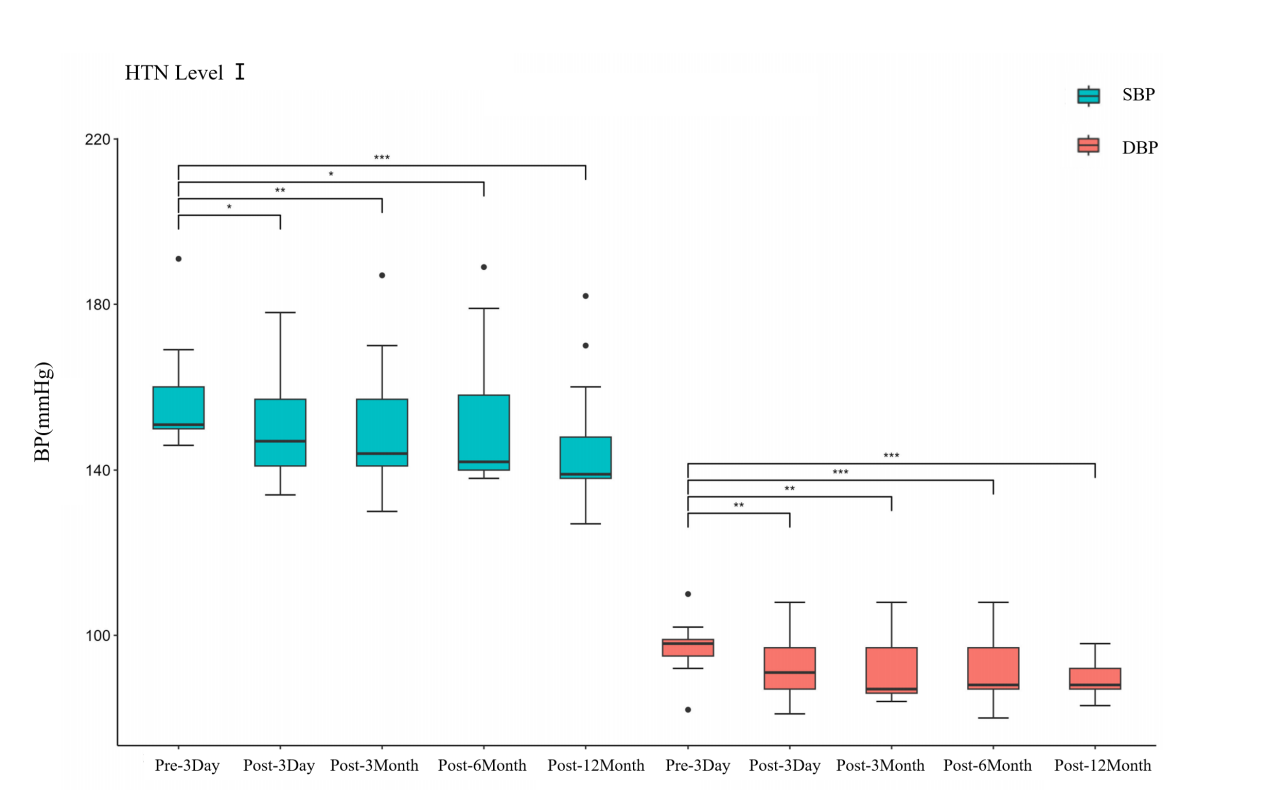


**Supplementary Figure 13.** Subgroup analysis-HTN Level I：Patients with different blood pressure classifications had a significant decrease in blood pressure after MVD (P<0.05), and there was a sustained antihypertensive effect in all of them. The higher the hypertension classification, the higher the preoperative blood pressure, the greater the decrease in blood pressure after MVD, and the systolic blood pressure was controlled at the level of about 150 mmHg and diastolic blood pressure was controlled at the level of about 100 mmHg, as shown in Figures 13, 14 and 15.


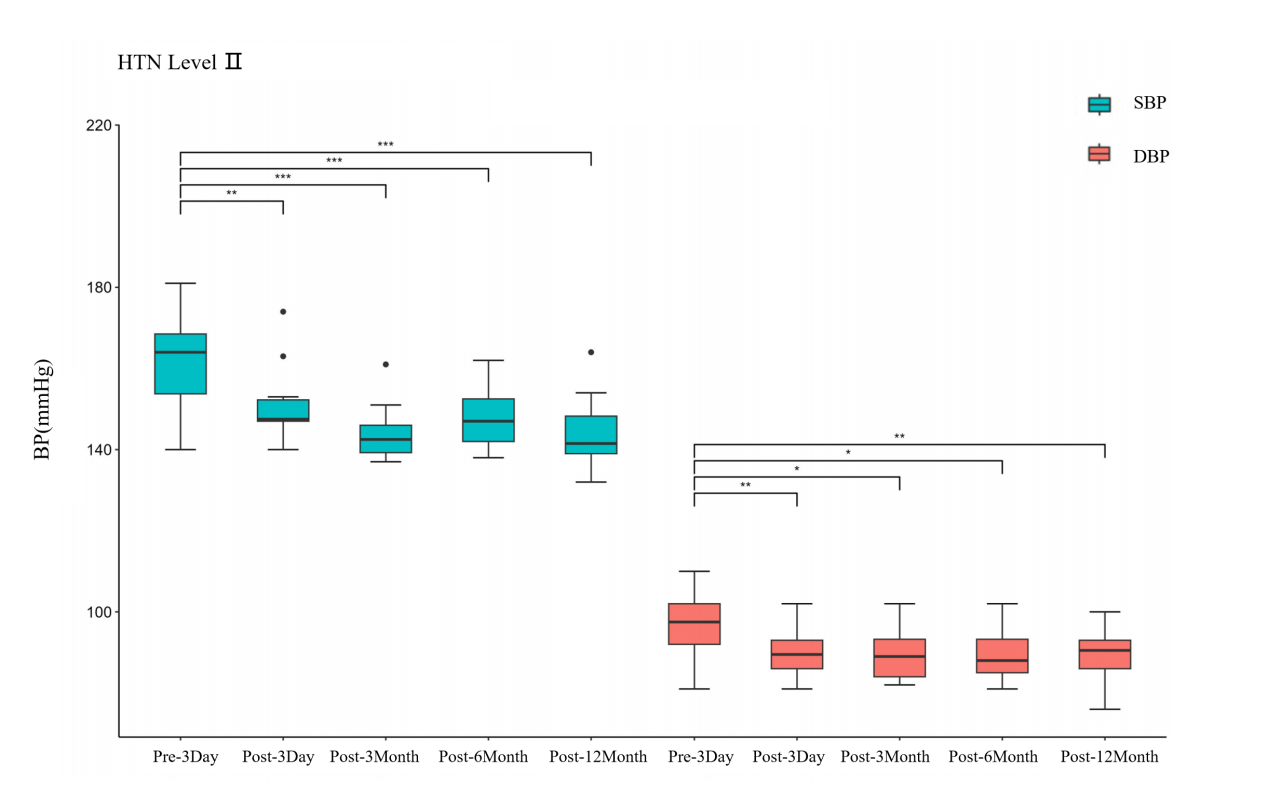


**Supplementary Figure 14.** Subgroup analysis-HTN Level Ⅱ：Patients with different blood pressure classifications had a significant decrease in blood pressure after MVD (P<0.05), and there was a sustained antihypertensive effect in all of them. The higher the hypertension classification, the higher the preoperative blood pressure, the greater the decrease in blood pressure after MVD, and the systolic blood pressure was controlled at the level of about 150 mmHg and diastolic blood pressure was controlled at the level of about 100 mmHg, as shown in Figures 13, 14 and 15.


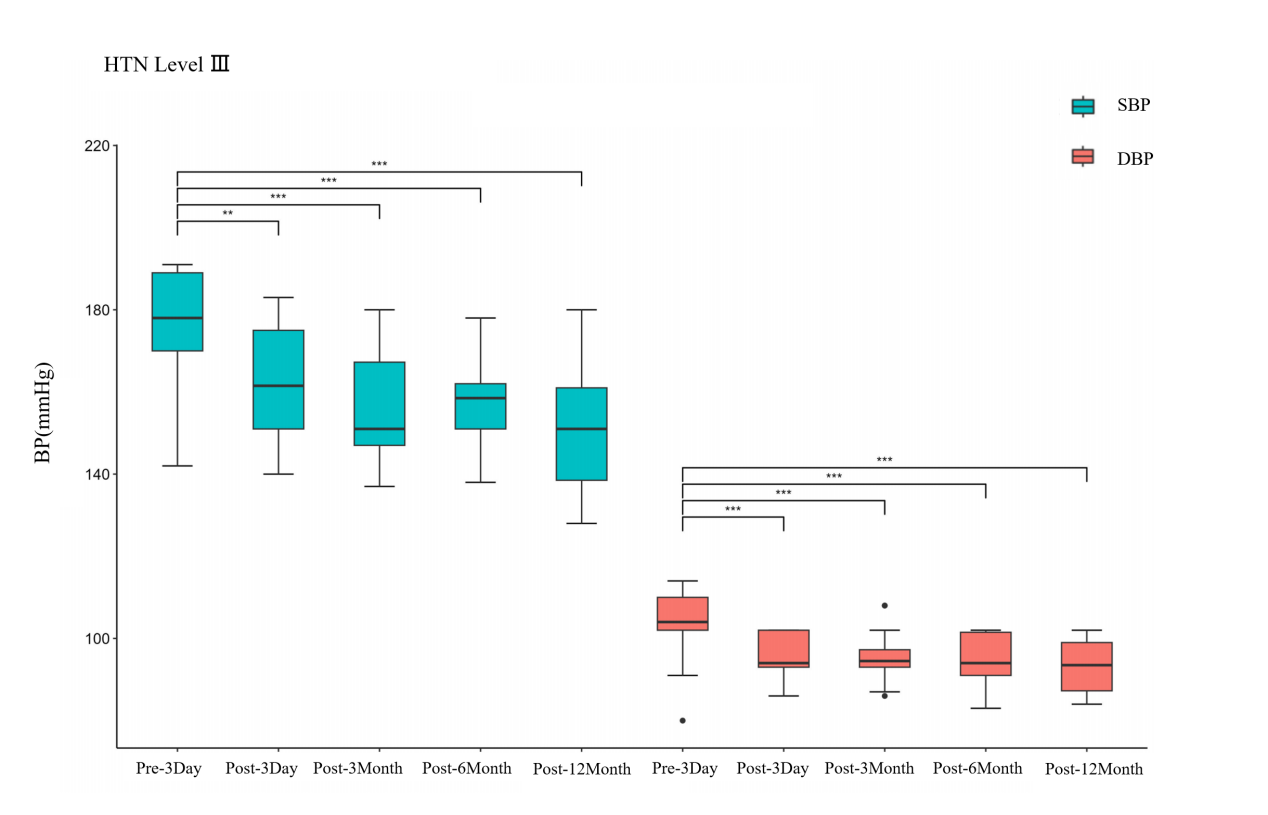


**Supplementary Figure 15.**Subgroup analysis-HTN Level Ⅲ：Patients with different blood pressure classifications had a significant decrease in blood pressure after MVD (P<0.05), and there was a sustained antihypertensive effect in all of them. The higher the hypertension classification, the higher the preoperative blood pressure, the greater the decrease in blood pressure after MVD, and the systolic blood pressure was controlled at the level of about 150 mmHg and diastolic blood pressure was controlled at the level of about 100 mmHg, as shown in Figures 13, 14 and 15.


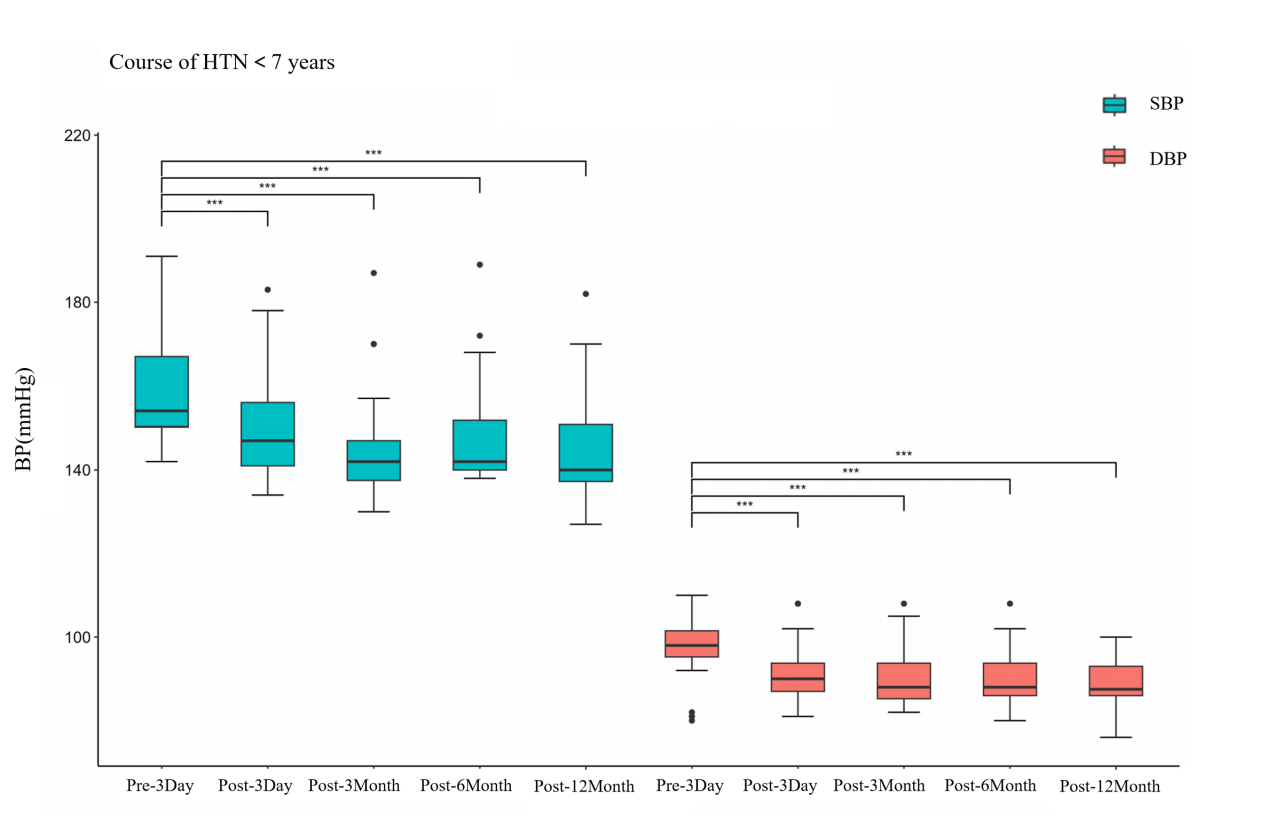


**Supplementary Figure 16.** Subgroup analysis-Course of HTN ＜ 7 years：Patients were divided into two groups using the median history of hypertension (7 years) as the splitting point. patients with a longer history of hypertension had higher preoperative blood pressure levels than patients with a shorter history of hypertension and had a greater decrease in systolic blood pressure, as shown in Figures 15 and 16. patients with different histories of hypertension had a significant decrease in blood pressure after MVD (P<0.05), and there was a sustained blood pressure-lowering effect in all of them.


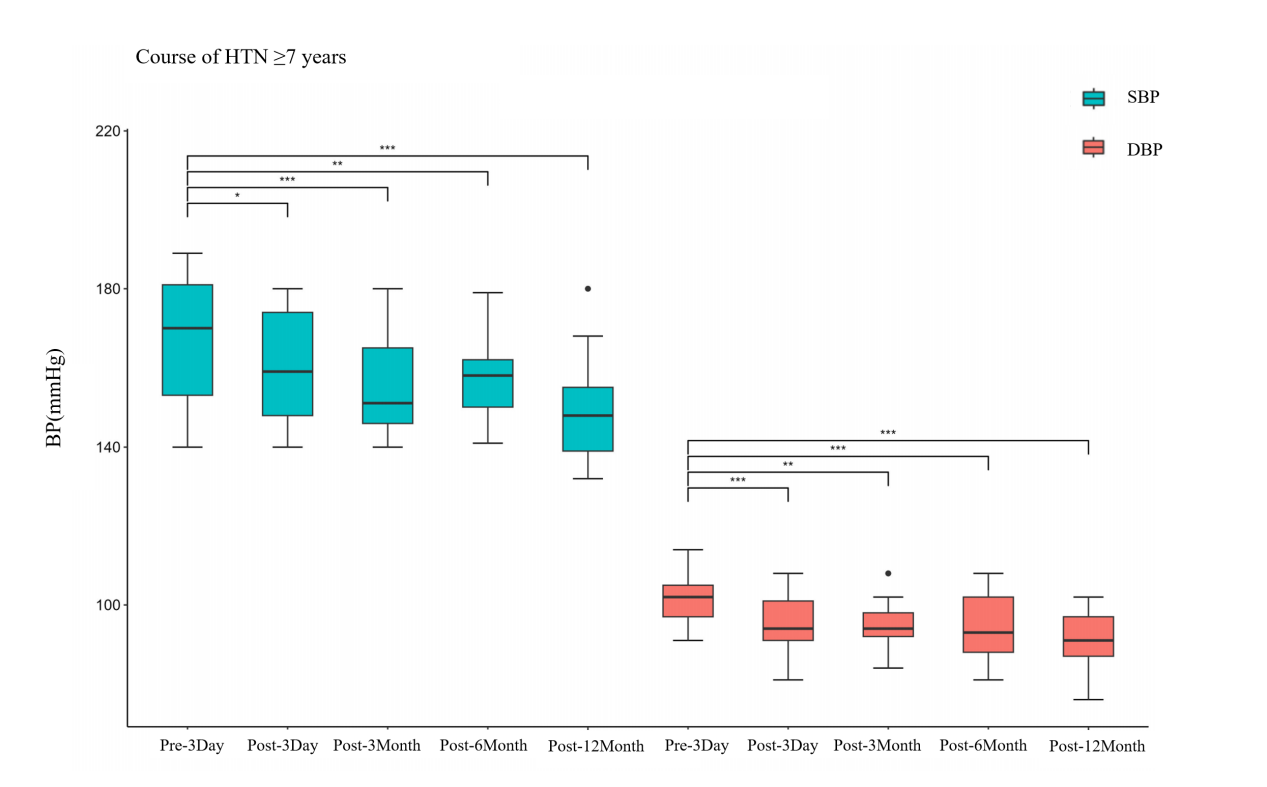


**Supplementary Figure 17.** Subgroup analysis-Course of HTN ≥7 years：Patients were divided into two groups using the median history of hypertension (7 years) as the splitting point. patients with a longer history of hypertension had higher preoperative blood pressure levels than patients with a shorter history of hypertension and had a greater decrease in systolic blood pressure, as shown in Figures 16 and 17. patients with different histories of hypertension had a significant decrease in blood pressure after MVD (P<0.05), and there was a sustained blood pressure-lowering effect in all of them.









**Table 1.** The medication of 33 patients with effective hypertension after MVD
